# Effect of Rapid Thermal Annealing on Si-Based Dielectric Films Grown by ICP-CVD

**DOI:** 10.1021/acsomega.3c04997

**Published:** 2023-08-08

**Authors:** Irina Parkhomenko, Liudmila Vlasukova, Fadei Komarov, Nataliya Kovalchuk, Sergey Demidovich, Ainur Zhussupbekova, Kuanysh Zhussupbekov, Igor V. Shvets, Oleg Milchanin, Dmitry Zhigulin, Ivan Romanov

**Affiliations:** † Belarusian State University, Kurchatov Str. 5, 220045 Minsk, Belarus; ‡ A.N. Sevchenko Institute of Applied Physical Problems of Belarusian State University, Kurchatov Str. 7, 220045 Minsk, Belarus; § Joint Stock Company “Integral”, Kazintsa Str. 121 A, 220108 Minsk, Belarus; ∥ School of Physics and Centre for Research on Adaptive Nanostructures and Nanodevices (CRANN), Trinity College Dublin, Dublin D02 PN40, Ireland; ⊥ L.N. Gumilyov Eurasian National University, 2 Satpayev Street, Astana 010000, Kazakhstan

## Abstract

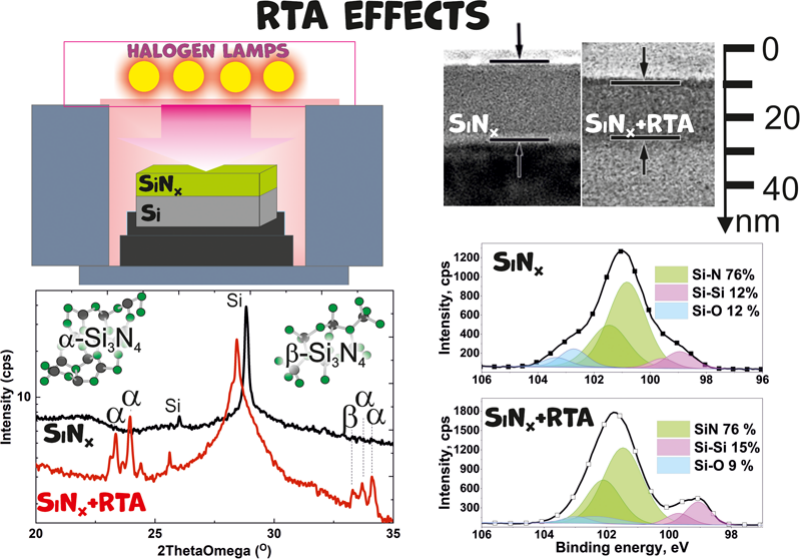

Silicon nitride, silicon oxide, and silicon oxynitride thin films were deposited on the Si substrate by inductively coupled plasma chemical vapor deposition and annealed at 1100 °C for 3 min in an Ar environment. Silicon nitride and silicon oxide films deposited at ratios of the reactant flow rates of SiH_4_/N_2_ = 1.875 and SiH_4_/N_2_O = 3, respectively, were Si-rich, while Si excess for the oxynitride film (SiH_4_/N_2_/N_2_O = 3:2:2) was not found. Annealing resulted in a thickness decrease and structural transformation for SiO*_x_* and SiN*_x_* films. Nanocrystalline phases of Si as well as α- and β-Si_3_N_4_ were found in the annealed silicon nitride film. Compared to oxide and nitride films, the oxynitride film is the least susceptible to change during annealing. The relationship between the structure, composition, and optical properties of the Si-based films has been revealed. It has been shown that the calculated optical parameters (refractive index, extinction coefficient) reflect structural peculiarities of the as-deposited and annealed films.

## 1 Introduction

Despite all the talk of the end of the silicon era, the Si-based semiconductor industry will continue to satisfy the needs of the market. Recently, much attention has been paid to silicon-based dielectrics such as SiO*_x_*, SiN*_x_*, and SiO*_x_*N*_y_*. Even more, silicon nitride and oxynitride are considered alternative dielectric platforms for integrated photonics.^1−3^

Nowadays, the progress of electronics is related to further miniaturization of chip technology. On-chip capacitor miniaturization requires a thickness decrease of basic dielectric layers such as silicon oxide and nitride films. In addition, there is a tendency to use layered structures and superlattices containing such thin dielectric films.^4−7^ Thin films of silicon nitride and oxide enriched with silicon are in the focus of interest today due to the possibility of using them as storage medium for resistive random-access memory (RRAM).^8−12^ To obtain the RRAM device with reliable and reproducible resistive switching characteristics, the thickness, chemical, and phase composition of storage medium—Si-based dielectric layer—should be strictly controlled. Unfortunately, control and diagnostics of ultrathin Si-based dielectric layers are getting dramatically more complicated.

In this work, we investigated thin dielectric films of Si-enriched silicon oxide and nitride as well as silicon oxynitride obtained by inductively coupled plasma chemical vapor deposition (ICP-CVD). This low-temperature method allows us to deposit uniform large-area films with a low level of radiative defects, which is crucial for modern technologies including conformable electronics.^13^ To the best of our knowledge, Si-based ICP-CVD films have not been studied in detail, especially oxide and oxynitride ones. Also, the effect of rapid thermal annealing of ICP-CVD Si-based films is scarcely represented in the published literature. Besides, the ICP-CVD films with a thickness of more than 60 nm were mostly discussed in published articles.^13−16^ The thickness of the films discussed in the present work is about 10–25 nm. Diagnostics of such thin films is more complicated, yet necessary, as the role of interface effects during deposition and subsequent annealing increases with decreasing thickness.^17^ The aim of our study was to reveal the relationship between the structure and composition and the optical properties of the Si-based films, all immensely important for the future design of optoelectronic and photonic systems based on such films.

## 2 Experimental Section

Silicon nitride, oxide, and oxynitride films were deposited on p-type silicon substrates ⟨111⟩ (100 mm in diameter) by ICP-CVD using a STE ICP200D system (SemiTEq). The initial silicon substrates were treated in Caro’s acid and ammonia peroxide mixture and further cleaned in Ar in the reactor at an ICP source power of 300 W for 120 s at 105 °C. The deposition temperature in all cases was 300 °C, and the RF discharge power was in the range of (800–1000) W. The flow rate of reagent gases, chamber pressure, deposition time (*t*), and deposition rate (*r*) are given in Table 1. The flow rate of monosilane (SiH_4_) remained the same for all deposition processes. Nitrogen (N_2_) and nitrous oxide (N_2_O) were used as reactant gases for the deposition of SiN*_x_* and SiO*_x_*, respectively. In the case of SiO*_x_*N*_y_*, both these gases mixed in equal proportions were utilized. An Ar + He mixture was used as a carrier gas.

**Table 1:** Deposition Regimes of Si-Based Dielectric Films

sample	SiH_4_ (sccm)	N_2_ (sccm)	N_2_O (sccm)	Ar (sccm)	He (sccm)	*p* (Pa)	ICP power (W)	*t* (s)	*r* (nm/min)
SiN*_x_*	15	8		75	120	2.5	1000	37	39.6
SiO*_x_*	15		5	10	120	1.8	800	12	58.5
SiO*_x_*N*_y_*	15	10	10	40	120	2.0	1000	41	34.4

Then, the 1 × 1 cm^2^ samples were cut out from wafers with deposited dielectric films, placed on a silicon substrate, and annealed at 1100 °C for 3 min in an Ar atmosphere using a rapid thermal annealing (RTA) furnace (AS-Master, Annealsys, France) with a heating rate of 70 °C/s.

The thicknesses of layers were measured by scanning electron microscopy (SEM) using a Hitachi S-4800 microscope and by transmission electron microscopy (TEM) in a cross-sectional technique using a Hitachi H-800 microscope operated at 200 keV. Additionally, TEM images in a plan-view technique and selected area electron diffraction (SAED) patterns were taken.

The crystal structure and compositional analyses of the ultrathin films were performed via X-ray diffraction (XRD) and X-ray photoelectron spectroscopy (XPS), respectively. The elemental composition was measured by XPS in an Omicron MultiProbe XPS instrument (Scienta Omicron Inc., Uppsala, Sweden). High-resolution spectra were obtained at 20 eV pass energy using a monochromatic X-ray source. The obtained spectra were analyzed using CasaXPS software with fitting of the Gauss–Lorentz form and the Shirley background. All energy positions are corrected for C 1*s* (284.8 eV). Photoelectronic spectra of Si 2p levels were fitted by three components (Si–N, Si–O, elemental Si). Each component was adjusted taking into account the contributions of Si 2p_3/2_ and Si 2p_1/2_^18^ and the spin–orbit splitting of 0.63 eV, usually employed for elemental Si. XRD measurements were performed on an X-ray diffractometer (Bruker d8 Advance) using a un-monochromated Cu Kα radiation (wavelength of 1.54 Å) at 40 kV and 40 mA through a 0.6 mm slit at an angle of (10–70°).

The optical properties were investigated through measurements of the specular reflectance spectra at 8° incident angle using the universal reflectance accessory of a LAMBDA-1050 ultraviolet–visible–near-infrared(UV–vis–NIR) spectrophotometer in the range of 190–1000 nm with an accuracy of 0.1%. RefFit Software^19^ was used to fit the reflectance spectra and to extract optical parameters (refractive index, extinction and absorption coefficient, energy band gap).

## 3 Results and Discussion

### 3.1 SEM and TEM

**Figure 1:**
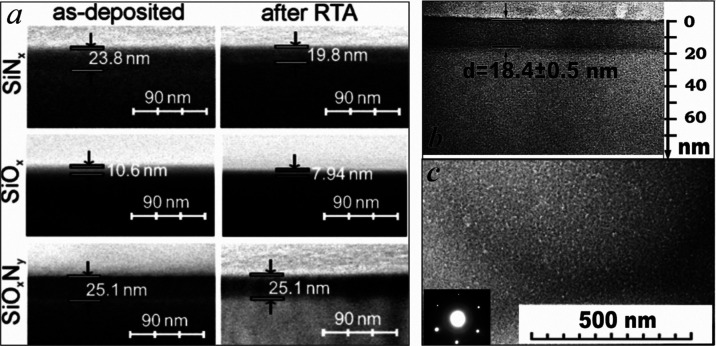
Cross-sectional SEM images of the SiN*_x_*, SiO*_x_*, and SiO*_x_*N*_y_* films before and after RTA (a) and cross-sectional (b) and dark-field plan-view (c) TEM images of the SiN*_x_* film after RTA. The inset shows the corresponding SAED pattern.

In order to obtain reliable information from optical spectra (refractive index, extinction coefficient), it is necessary to know the exact value of the film thickness. In this work, the thickness of the as-deposited films was initially determined by ellipsometry. However, this method can give erroneous results for nonstoichiometric films. Therefore, the film thickness was additionally estimated by SEM and TEM. The results of thickness measurements by various methods are shown in Table 2. For greater clarity, the cross-sectional SEM images of the samples with dielectric films are shown in Figure 1a. The corresponding TEM images are shown in Figure S1 of the SI.

**Table 2:** Thickness of Si-Based Dielectric Films

sample	ellipsometry *d* (nm)	SEM *d* (nm)	TEM *d* (nm)
SiN*_x_*	24.4	23.8 ± 1	24.6 ± 2
SiN*_x_* after RTA		19.8 ± 1	18.4 ± 0.5
SiO*_x_*	11.7	10.6 ± 1	11.9 ± 1.0
SiO*_x_* after RTA		7.9 ± 1	7.5 ± 0.5
SiO*_x_*N*_y_*	23.5	25.1 ± 1	26.9 ± 1.0
SiO*_x_*N*_y_* after RTA		25.1 ± 1	28.8 ± 1.0

As can be seen from Table 2, a good correlation is observed between the values of thicknesses obtained by different methods. Besides, SEM and TEM data reveal the thickness decrease for the SiN*_x_* and SiO*_x_* films after thermal treatment. Probably, this is due to the film densification and the increase in residual stress after annealing.^20^ In the case of SiO*_x_*N*_y_*, SEM data have not changed, while TEM data show a slight increase of thickness. The absence of the film shrinkage indicates the suppression of increasing residual stress during the heat treatment.

Figure 1b,c shows the cross-sectional and dark-field plan-view TEM images of the annealed SiN*_x_* film, respectively. The dark-field TEM image displays small inclusions of light contrast, which indicates the presence of crystalline nanoclusters. Unfortunately, the SAED reflections from the silicon substrate suppress the weak signal from the nanocrystals.

### 3.2 XRD

**Figure 2:**
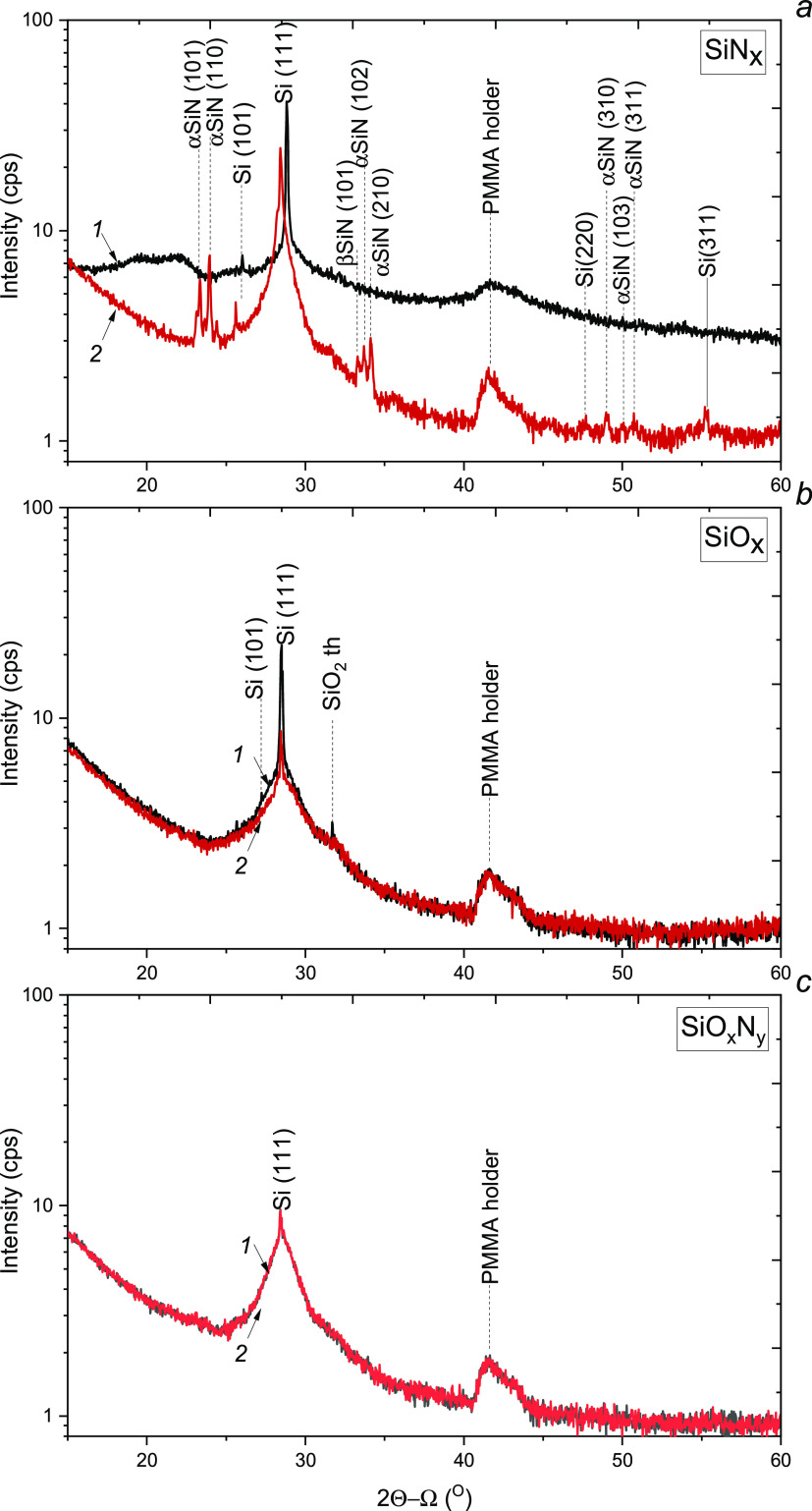
XRD spectra of the SiN*_x_* (a), SiO*_x_* (b), and SiO*_x_*N*_y_* (c) films before (1) and after RTA (2).

Figure 2 depicts the XRD spectra of the samples.

A peak at around 28.5° is observed in all spectra. This peak is attributed to the Si crystal facets of (111) and is due to the substrate contribution.^21,22^ However, in the case of the sample with the as-deposited SiN*_x_*, a shift of this peak position to 28.8° is observed. It can indicate a tensile strain of Si. We suppose that it is due to formation of the surface transition silicon layer at the initial step of deposition. Afterward, this transition layer crystallized and cooled testing tensile stress under the top SiN*_x_* layer. The similar effect of the thin capping layer on residual stress underlying silicon is described in refs (23, 24). The wide band at 20–22° corresponding to the amorphous phase should be also noted, presumably SiO*_x_*.^25,26^ RTA eliminates the “tensile strain” shift and “amorphous” band. Besides, RTA results in broadening of the (111) peak as well as formation of a narrow peak set. These narrow peaks indicate the formation of Si nanocrystals^27−29^ and nanocrystalline phases, α- and β-Si_3_N_4_.^22^ Formation of such phases occurs at normal pressure yet at high temperature.^30^ However, these requirements for nanocrystals can be relaxed.^31^ For example, α-Si_3_N_4_ nanocrystals were registered in silicon oxynitride films deposited using ICP-CVD.^14^ The multilayered plasma-enhanced CVD-SiN*_x_* film contained α- and β-Si_3_N_4_ nanocrystals after annealing at 1100–1150 °C.^22,31^ Moreover, the 2 × 2 silicon (111) unit cell has a lattice mismatch of only about 1.1% with the “*a*” axis of hexagonal Si_3_N_4_.^32^ Therefore, the orientation of the Si substrate (111) in our case favors the formation of the silicon nitride crystalline phase. Thus, we can conclude that clusters with light contrast observed in the TEM image (Figure 1c) are Si and Si_3_N_4_ nanocrystals.

The effect of RTA on the crystal structure is less expressed for the silicon oxide film. In the case of the as-deposited SiO*_x_* film, the Si (111) band is wider than for the as-deposited silicon nitride. This broadening can be related to the amorphous silicon or silicon oxide phase. RTA results in this band narrowing that indicates crystallization of the amorphous Si phase and/or ordering of the SiO network. The as-deposited silicon oxynitride sample also demonstrates broadening of the (111) band in comparison with the SiN*_x_* one; however, RTA does not result in noticeable changes.

### 3.3 XPS

The atomic concentrations of the elements Si, C, N, and O for the SiN*_x_*, SiO_x_, and SiO*_x_*N*_y_* films calculated from XPS data are shown in Table 3. Taking into account the stoichiometric ratio for silicon nitride ([N]/[Si] = 1.3) and silicon oxide ([O]/[Si] = 2), the as-deposited SiN*_x_* and SiO*_x_* films are Si-rich ones. The Si excess is 34 and 57% for SiN*_x_* and SiO*_x_*, respectively. In the case of silicon oxynitride films, the ratio [O]/([O] + [N]) was calculated. The value of this parameter below/above 0.4 indicates a nitride-like/oxide-like structure.^33^ In our case, the as-deposited SiO*_x_*N*_y_* film exhibits a ratio of 0.6 that suggests an oxide-like structure. RTA results in the decrease of carbon contamination for all films. In the case of SiN*_x_* and SiO*_x_*N*_y_*, the oxygen concentration increases, while for the SiO*_x_* film, the nitrogen concentration increases after RTA.

**Table 3:** Atomic concentration (atom %) of Si, N, C, and O Elements of Si-Based Dielectric Films

sample	Si	N	O	C	atomic ratio
SiN*_x_*	51.4	17.0	12.8	21.6	[N]/[Si]∼0.3
SiN*_x_* after RTA	50.5	20.8	16.4	13.3	[N]/[Si]∼0.4
SiO*_x_*	55.7	7.7	22.0	26.0	[O]/[Si]∼0.4
SiO*_x_* after RTA	44.9	12.4	22.3	10.2	[O]/[Si]∼0.5
SiO*_x_*N*_y_*	56.4	14.9	26.9	14.6	[O]/[O + N]∼0.6
SiO*_x_*N*_y_* after RTA	54.05	12.45	28.1	13.5	[O]/[O + N]∼0.7

Let us briefly discuss the presence of hydrogen in the films under investigation. We failed to register the signal from hydrogen or Si–H and N–H bonds by XPS as well as by IR spectroscopy (not shown here). Possibly, the films in our experiment are too thin, and the H-signal is below the detection limit of using techniques. Nevertheless, we are inclined toward the low hydrogen concentration in the as-deposited dielectric films. First, ICP-CVD films are characterized as a low H content due to the used high-density plasma and N_2_ as a precursor instead of NH_3_.^34,35^ Second, one needs to consider the relatively high chosen value of deposition temperature and ICP power, which stimulates a reduction of H the content.^36^

**Figure 3:**
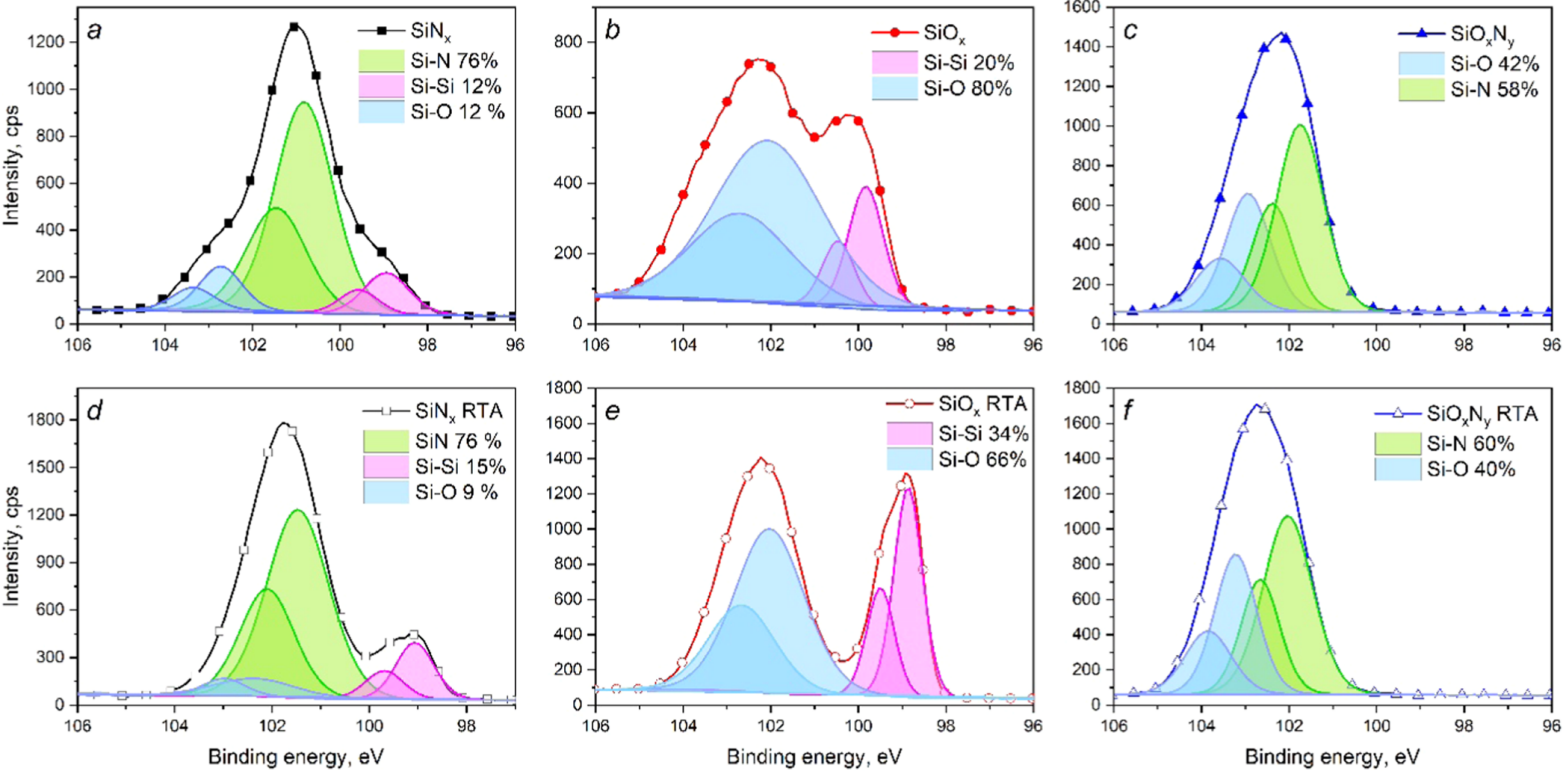
Si 2p XPS spectra of the SiN*_x_* (a, d), SiO*_x_* (b, e), and SiO*_x_*N*_y_* (c, f) films before (a–c) and after RTA (d–f).

Figure 3 shows the XPS spectra of the as-deposited and annealed Si-based dielectrics.

Quantitative analysis was carried out using the Si 2p core level. The range at 98–105 eV displays Si 2p peaks deconvoluted to Si^4+^, Si^3+^, Si^2+^, and Si^1+^ components related to dioxide and nitride phases and suboxide phases.^37,38^ The obtained results are presented in the insets in Figure 3. The XPS spectrum of the as-deposited SiN*_x_* film displays components assigned to silicon oxide, silicon nitride, and elemental silicon. The broad peaks imply a variation in atomic arrangements surrounding the bonds. Annealing results in a decrease of the contribution from Si–O bonds. The bands related to Si–N and Si–Si bonds become narrower and more defined, indicating the increase in the crystallization degree. It agrees with the XRD data on a decrease of contribution from the amorphous silicon oxide phase and about Si and Si_3_N_4_ nanocrystal formation.

Broad peaks related to Si–O and Si–Si bonds are manifested in the spectrum of the as-deposited SiO*_x_* film. RTA leads to narrowing of Si–O and increase of Si–Si components, as in the case of SiN*_x_*. A comparison of SiO*_x_* and SiN*_x_* samples reveals a higher percentage increase of the Si–Si band for SiO*_x_* films (by 14%). One can suggest that a formation of Si nanocrystals in the SiO*_x_* matrix during RTA proceeds more actively due to the higher Si excess in the as-deposited film. The absence of separate Si peaks in the XRD spectrum of the annealed SiO*_x_* sample, which were registered for SiN*_x_*, can be explained by the thinner (at least 2 times) film.

The XPS spectrum of the as-deposited oxynitride exhibits only bands related to Si–O and Si–N bonds and no bands from Si–Si bonds. Compared to oxide and nitride films, the oxynitride film is the least susceptible to change during RTA.

### 3.4 Optical Properties

**Figure 4:**
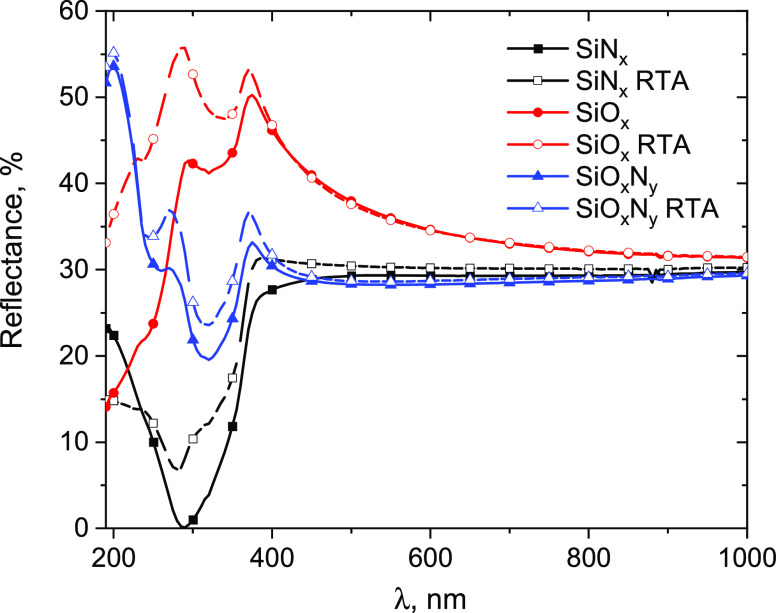
Reflectance spectra of the SiN*_x_*, SiO*_x_*, and SiO*_x_*N*_y_* films before and after RTA.

Figure 4 shows the reflectance spectra of the as-deposited and annealed films. The as-deposited SiN*_x_* film demonstrates reflectance minimum *R* = 0.087% at 290 nm. Such a low value of reflectance suggests that the refractive index of the SiN*_x_* film corresponds to almost perfect antireflection coating (square root of the substrate’s refractive index). RTA results in fading of the antireflection effect (*R* = 6.85%). In the case of the SiO*_x_* film, annealing also leads to the increase of reflectance by ∼10–20% in the range of 200–400 nm. The same trend, yet to a lesser extent, is observed for oxynitride films.

**Figure 5:**
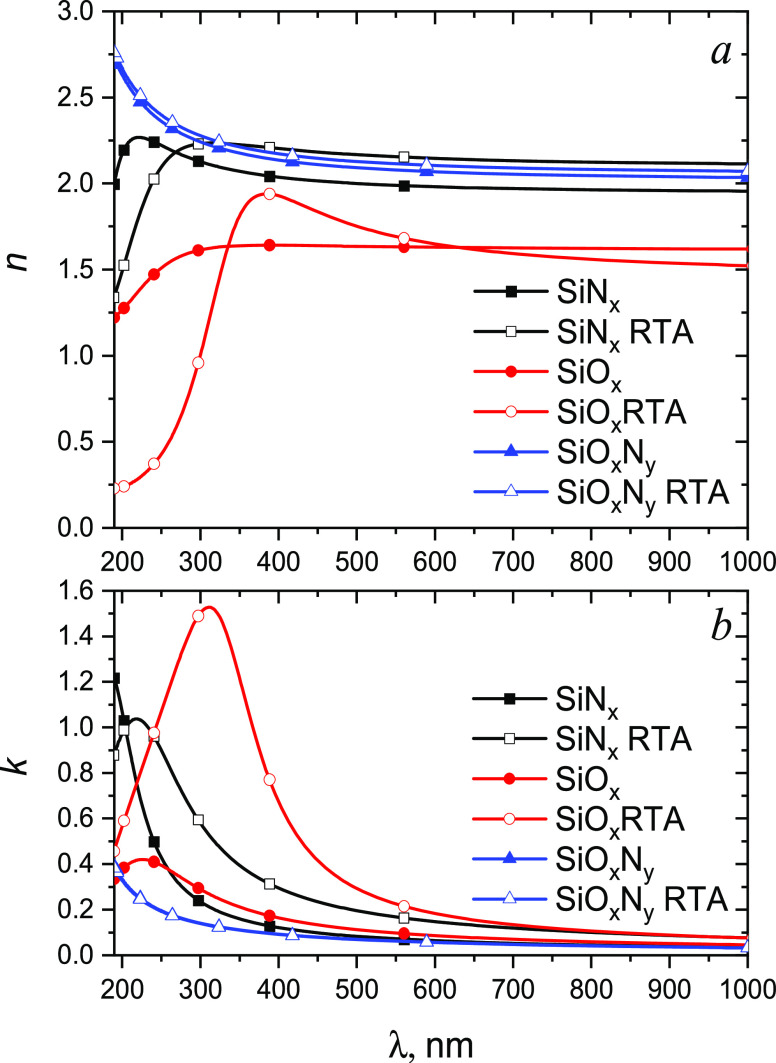
Dispersion of refractive index (a) and extinction coefficient (b) of the SiN*_x_*, SiO*_x_*, and SiO*_x_*N*_y_* films before and after RTA.

Figure 5 shows the corresponding refractive index *n* and extinction coefficient *k* as functions of wavelength obtained using RefFit software. The refractive index of the SiN*_x_* film is about 2.0–2.1 in the VIS range, typical for the CVD SiN*_x_* film.^15,39^ However, the refractive index spectrum of the as-deposited SiN*_x_* exhibits a maximum at 220 nm, and the extinction coefficient is rather high (0.05–0.015 in VIS). It is typical for Si-rich films.^40,41^ RTA results in an increase of *n* and *k* in the visible spectral range and a red-shifted maximum in the UV range. The red shift is closely related to an optical band gap decrease. According to our estimates using Tauc’s plots (Figure S2 in SI), the optical band gap decreases from 3.5 to 2.4 eV after RTA.

The refractive index and extinction coefficient of the SiO*_x_* film is also quite high, *n* ∼ 1.6 and *k* ∼ 0.07–0.23 in the VIS range, respectively. It indicates a high Si content in the film or/and high level of structural disordering^42^ that agrees with XPS data. As in the case of SiN*_x_*, RTA results in the refractive index increase with an appearance of a maximum at 385 nm and in a substantial increase of the extinction coefficient up to 1.5 at 310 nm. It additionally proves the synthesis of Si nanocrystals in the silica matrix.

The silicon oxynitride film exhibits the refractive index *n* = 2.05–2.15 in the VIS range that is close to *n* for silicon nitride. It can assume a low concentration of oxygen in the oxynitride film.^43^ The extinction coefficient (*k* ∼ 0.05–0.1) is the lowest among the discussed dielectrics. The obtained values of *n* and *k* are less than the ones for the Si-rich silicon oxynitride deposited by magnetron sputtering in ref (44) that additionally confirmed the absence of Si excess in the deposited film. Ref (45) reports the results of the study of SiN*_x_*O_y_ thin films deposited under different conditions at room temperature by plasma-enhanced CVD. The authors studied the chemical composition of SiN*_x_*O*_y_* films by FTIR spectroscopy and presented the N/Si ratio as a function of the refractive index. In our experiment, the silicon oxynitride film is characterized with *n* = 2.05–2.15. According to ref (45), this corresponds to the N/Si ratio ≈ 1.3. The values of *n* and *k* for SiO*_x_*N*_y_* remain virtually unchanged after RTA, which additionally confirms the stability of the film’s structure.

The similar behavior of optical constants for SiN*_x_* and SiO*_x_* films after annealing can be explained by the formation of Si-rich films with the disordering structure due to a relatively large flow rate of SiH_4_. Further annealing stimulates some structure transformation, namely atomic rearrangement of the host matrix as well as formation of nanocrystals. Such processes appear to be responsible for shrinking thicknesses for SiN*_x_* and SiO*_x_* films. In the case of silicon oxynitride, the flow rate of SiH_4_ is the same as for deposition of SiN*_x_* and SiO*_x_*. However, SiH_4_ molecules could interact with N_2_ as well as with N_2_O, and the Si excess in the deposited oxynitride film is minor. This favors the formation of a homogeneous structure resistant to high temperatures.

## 4 Conclusions

Silicon oxide, nitride, and oxynitride films with the thickness of 10–25 nm were synthesized by ICP-CVD at the fixed SiH_4_ flow rate. The other reactants of the deposition process were N_2_, N_2_O, and N_2_ + N_2_O for SiN*_x_*, SiO*_x_*, and SiO*_x_*N*_y_*, respectively. After that, the samples went through RTA at 1100 °C for 3 min. Practically important results are that RTA leads to a decrease of thickness for silicon oxide and nitride films, while the thickness of the silicon oxynitride film remains unchanged. We suggest that this is due to an excess content of Si and a disorder in the as-deposited SiN*_x_* and SiO*_x_* films, while the as-deposited SiO*_x_*N*_y_* has an initially more ordered structure without substantial Si excess. The rapid thermal annealing initiates structural transformation for SiN*_x_* and SiO*_x_* films, namely, recovery structure of the host matrix and formation of Si nanocrystals. Moreover, formation of α- and β-Si_3_N_4_ nanocrystals in the case of the SiN*_x_* film was revealed by XRD.

It has been shown that optical parameters (refractive index *n* and extinction coefficient *k*) reflect structural peculiarities of the as-deposited and annealed films. In the case of SiN_x_ and SiO*_x_*, RTA results in an increase of *n* and *k* in the VIS range as well as the maximum red shift in the UV range, which is typical for Si-rich films and indicates formation of Si nanocrystals in the dielectric matrix. The intensity of *n* and *k* maxima is higher in the spectra of SiO*_x_* samples. Hence, we can conclude that Si nanocrystal formation in the SiO*_x_* matrix proceeds more actively than in silicon nitride. It agrees with the XPS data. The revealed regularities can be useful in choosing Si-based dielectrics for electro-optics and MEMS devices. Also, formation of the crystalline silicon nitride film on the Si substrate can ensure the sustainability of crystalline growth of III nitrides materials (GaN, AlN) on silicon.

## Supporting Information Available

Additional cross-sectional TEM images of the SiN*_x_*, SiO*_x_*, and SiO*_x_*N*_y_* films as well as Tauc’s plot of SiN*_x_* before and after RTA is included (PDF)

## Abbreviations

RRAM: resistive random-access memory
ICP-CVD: inductively coupled plasma chemical vapor deposition
RTA: rapid thermal annealing
SEM: scanning electron microscopy
TEM: transmission electron microscopy
SAED: selected area electron diffraction
XPS: X-ray photoelectron spectroscopy
