# Supplementary material for: Effect of Rapid Thermal Annealing on Si-Based Dielectric Films Grown by ICP-CVD
Source: ACS Omega. 2023 Aug 8;8(33):30768–75. doi: 10.1021/acsomega.3c04997 (PMC10448691; doi:10.1021/acsomega.3c04997)
Supplement: Supplementary file 1 — ao3c04997_si_001.pdf [file ao3c04997_si_001.pdf]

Supporting Information

Effect of rapid thermal annealing on Si-based dielectric films grown by ICP-CVD

Irina Parkhomenko[a], Liudmila Vlasukova[a], Fadei Komarov[b], Nataliya Kovalchuk[c], Sergey Demidovich[c], Ainur Zhussupbekova[d,e], Kuanysh Zhussupbekov[d], Igor V. Shvets[d], Oleg Milchanin[b], Dmitry Zhigulin[c], Ivan Romanov[a]*

[a]Belarusian State University, Kurchatov Str. 5, 220045, Minsk, Belarus

[b]A.N. Sevchenko Institute of Applied Physics Problems, Kurchatov Str. 7, 220045, Minsk, Belarus

[c]Joint Stock Company "Integral", Kazintsa Str. 121 A, 220108, Minsk, Belarus

[d]School of Physics and Centre for Research on Adaptive Nanostructures and Nanodevices (CRANN), Trinity College Dublin, Dublin, Ireland

[e]L.N. Gumilyov Eurasian National University, 2 Satpayev Street, Astana, 010000 Kazakhstan

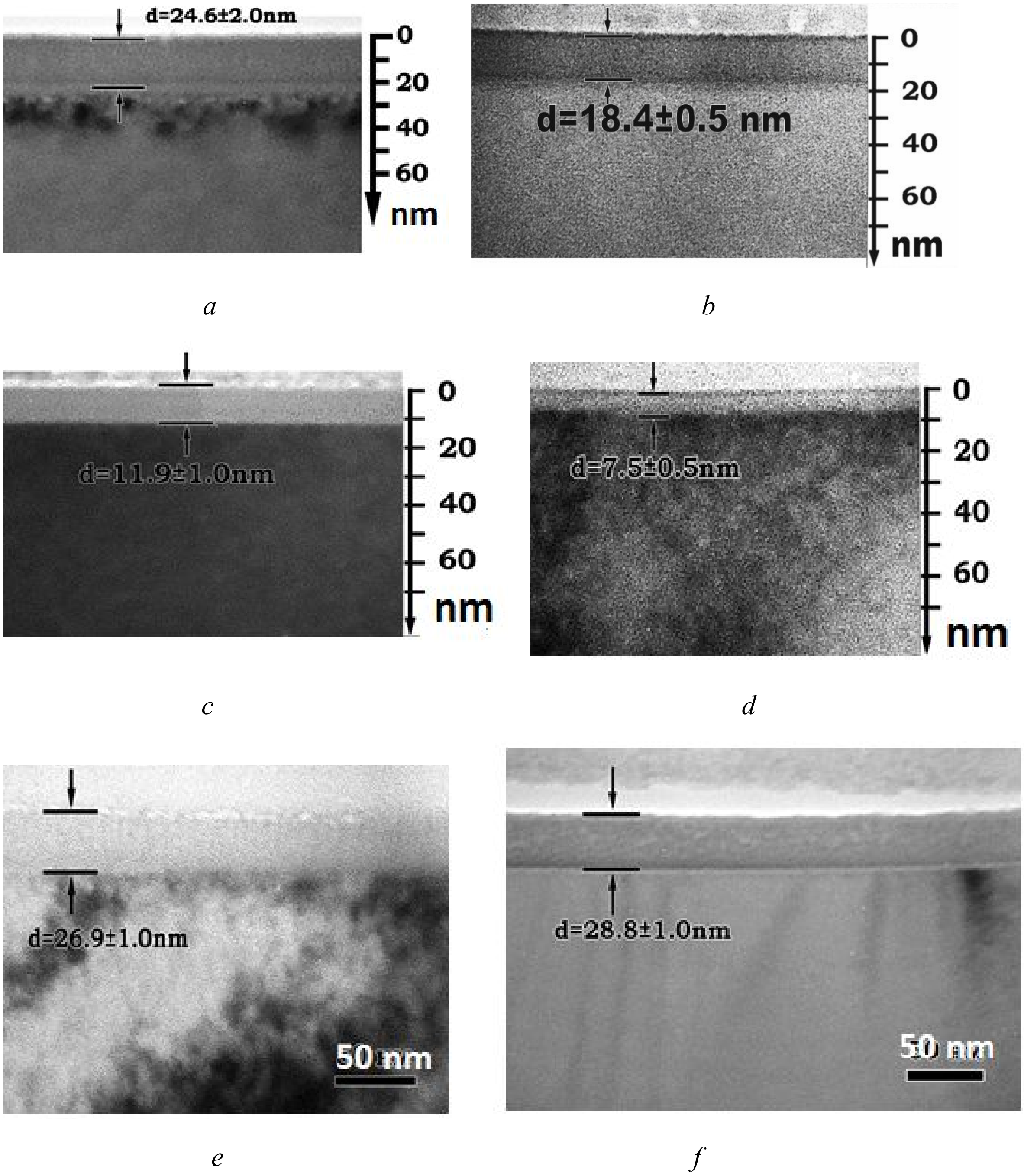

Fig. S1. Cross section TEM images of the SiN_x *(a,b)*, SiO_x (*c, d*) and SiO_xN_y (*e, f*) films before (*a, c, e*) and after RTA (*b, d, f*)

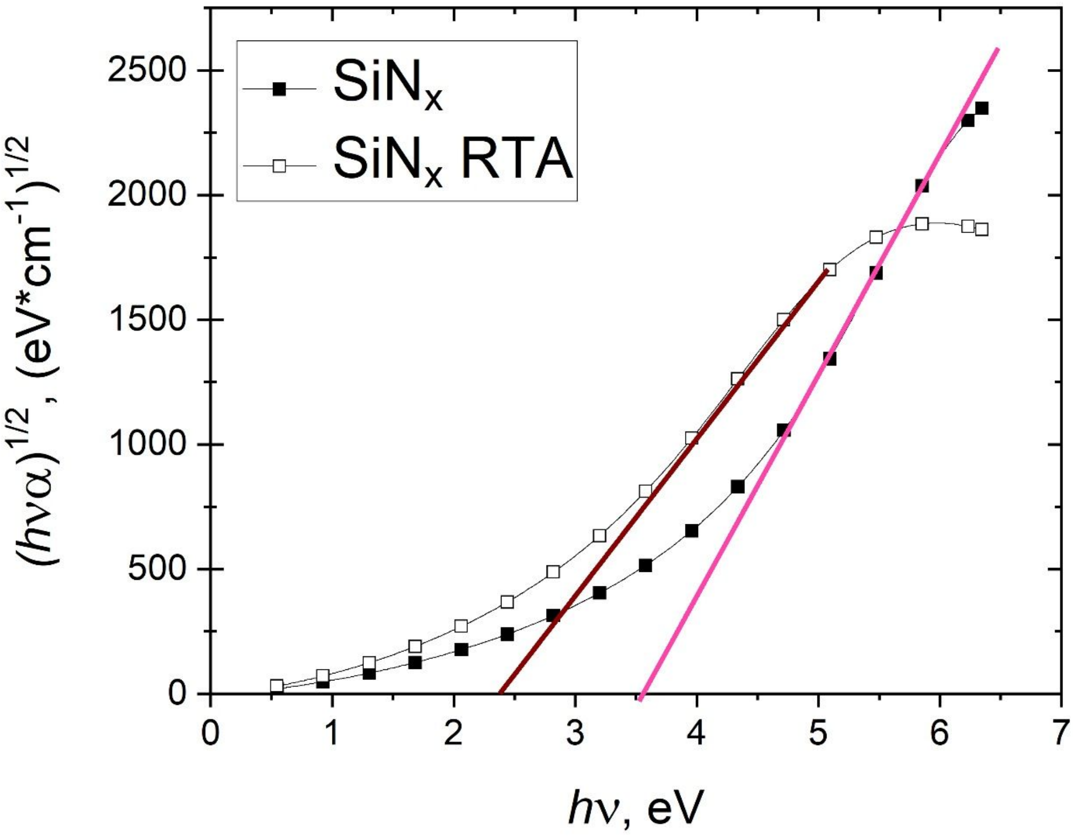

Fig. S2. Tauc's plot of the SiN_x before and after RTA
